# Magnetic Resonance Imaging and Histopathological Aspects of Botryomycosis

**DOI:** 10.1590/0037-8682-0646-2021

**Published:** 2022-02-25

**Authors:** Diogo Goulart Corrêa, Leonardo Hoehl Carneiro, Flavia Martins Costa

**Affiliations:** 1 Clínica de Diagnóstico por Imagem/DASA, Departamento de Radiologia, Rio de Janeiro, RJ, Brasil.; 2 Universidade Federal Fluminense, Departamento de Radiologia, Niterói, RJ, Brasil.; 3 Universidade Federal do Rio de Janeiro, Departamento de Patologia, Rio de Janeiro, RJ, Brasil.; 4 Universidade Federal do Rio de Janeiro, Departamento de Radiologia, Rio de Janeiro, RJ, Brasil.

**FIGURE 1:**
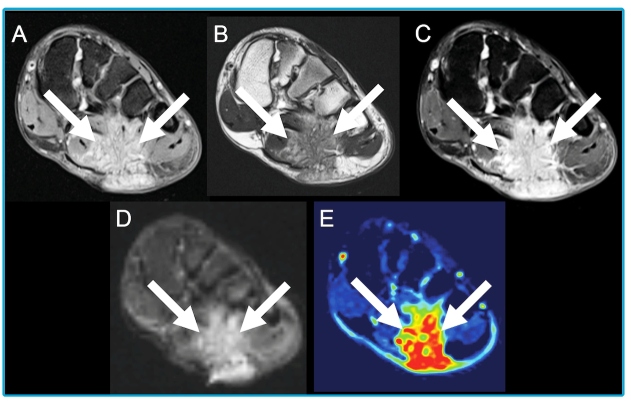
MRI of the left foot showing a large mass comprising multiple tiny clustered oval lesions affecting the left foot plantar soft tissues and infiltrating the flexor digitorum brevis and flexor hallucis brevis muscles and plantar fascia. The mass had a predominantly isointense signal on T1-weighted image with fat saturation (arrows in A) and hypointense signal on the T2-weighted image (arrows in B) due to the associated surrounding fibrous reaction; it showed heterogeneous gadolinium-enhancement (arrows in C). The lesion also presented restricted diffusion due to hypercellularity (arrows in D), and increased perfusion in its solid portions (arrows in E).

**FIGURE 2:**
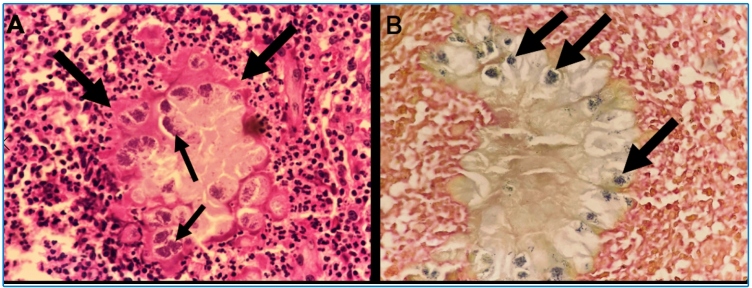
Histopathological analysis revealed extensive suppurative necrosis with fibrosis, associated with an eosinophilic coating (thick arrows in A), surrounding bacterial granules (thin arrows in A), characteristic of the Splendore-Hoeppli phenomenon (original magnification, × 200). The bacterial granules were Gram-positive (arrows in B; original magnification, × 400).

A 20-year-old woman presented with a growing mass on the plantar surface of the left foot that had been present for one year. She reported laceration trauma in this region six years earlier. The wound healed initially with topical antibiotics, but eventually, a palpable mass appeared. The patient was afebrile, had no skin discharge, and her hemogram was normal. Magnetic resonance imaging (MRI) demonstrated multiple clustered oval lesions affecting the soft tissues of the left foot, with heterogeneous gadolinium enhancement (Figure 1). After surgical resection, histopathological analysis revealed suppurative necrosis with fibrosis and an eosinophilic coating surrounding several gram-positive bacterial granules (Figure 2). *S. aureus* grew in the tissue culture. Cutaneous botryomycosis was diagnosed, and oral clindamycin was initiated.

Botryomycosis is a chronic suppurative bacterial infection that generally affects the skin and subcutaneous tissues. It has an insidious course, usually involving the extremities, and is associated with previous inoculating trauma, alcoholism, diabetes mellitus, and/or immunosuppression^1^ ^,^ ^2^. It can cause extensive destruction and disfigurement, associated with non-healing ulcers, sinuses, and fistulae. *S. aureus* is most commonly implicated. Botryomycosis is a misnomer still used for historical reasons^1^.

The MRI appearance of botryomycosis has rarely been described^2^; however, the presence of multiple rounded or oval clustered lesions in a limb with previous trauma should lead to suspicion of botryomycosis. Differential diagnoses include mycetoma and skin tumors^2^. Histopathology can demonstrate the Splendore-Hoeppli phenomenon, but it is not pathognomonic, as it occurs in other infectious and non-infectious diseases^3^. Thus, a culture of biopsied tissue is fundamental for diagnosis^1^ ^-^ ^3^.
